# Pathologic complete response of advanced hepatoid adenocarcinoma of the stomach following immuno-chemotherapy and conversion surgery: a rare case report and review of the literature

**DOI:** 10.3389/fonc.2025.1648766

**Published:** 2025-10-02

**Authors:** Yaoqi Li, You Wang, Tao Yu, Dong Wang, Hong Ma, Jichun Ma, Mingxu Da

**Affiliations:** ^1^ Department of Surgical Oncology, Gansu Provincial Hospital, Lanzhou, China; ^2^ The First School of Clinical Medicine, Gansu University of Traditional Chinese Medicine, Lanzhou, China; ^3^ Department of General Surgery, Chengxian People’s Hospital, Longnan, China; ^4^ Department of Medical Oncology, Liangzhou Hospital, Wuwei, China; ^5^ Department of Interventional Oncology, Gansu Provincial Hospital, Lanzhou, China

**Keywords:** HAS, pCR, immunotherapy, chemotherapy, conversion surgery

## Abstract

**Background:**

Hepatoid adenocarcinoma of the stomach (HAS) is a rare subtype of gastric cancer (GC) characterized by alpha-fetoprotein (AFP) production and invasive liver and lymph node metastases, typically associated with a poor prognosis. Although immuno-chemotherapy has made significant achievements in the conversion therapy of advanced GC in recent years, the management of HER2-negative, proficient mismatch repair (pMMR), and a programmed cell death ligand-1 (PD-L1) combined positive score (CPS)<5 cases, particularly in the context of synchronous multiple liver metastases and lymph node involvement, poses significant challenges. This is attributable not only to its rapid progression but also to its poor prognosis. We retrospectively report a case of HAS with concurrent multiple liver and lymph node metastases. Following six cycles of immuno-chemotherapy, R0 resection was achieved, and postoperative pathological examination confirmed a pathological complete response (pCR). No recurrence or metastasis was observed at the 32-month postoperative follow-up (last follow-up: April 26, 2025). To our knowledge, no previous reports have documented pCR in HER2-negative, pMMR, and PD-L1 CPS<5 patients with advanced HAS following conversion therapy with combined immuno-chemotherapy. This report aims to provide further clinical reference for the treatment of advanced HAS.

**Case summary:**

A 51-year-old male patient was diagnosed with HAS accompanied by multiple liver and lymph node metastases. Following six cycles of immunotherapy (sintilimab) combined with chemotherapy (Nab-paclitaxel, oxaliplatin, and S-1), the primary tumor exhibited significant reduction. Multiple liver metastases showed partial shrinkage or disappearance (the target lesion diameter must be less than 10 mm), and retroperitoneal lymph nodes were no longer detectable. After thorough evaluation, R0 resection was deemed achievable. Therefore, radical distal gastrectomy with D2 lymphadenectomy and liver metastasectomy were performed. Postoperative pathology confirmed pCR. The patient has remained progression-free survival (PFS) for 32 months and overall survival (OS) for 38 months, with no evidence of recurrence or metastasis.

**Conclusion:**

HAS is a highly invasive malignant tumor of the stomach. The dynamic changes in AFP serve as a reliable indicator for detecting HAS, evaluating treatment efficacy, and predicting recurrence. In advanced HER-2-negative, PD-L1 CPS<5, pMMR-type HAS, employing a conversion therapy regimen combining sintilimab with Nab-paclitaxel, oxaliplatin, and S-1 may reduce tumor staging, enhance conversion therapy success rates, and prolong survival.

## Introduction

GC is the fifth leading cause of cancer-related mortality worldwide, with approximately one million new cases reported annually (1). Epidemiological data indicate that China accounts for approximately 40% of global GC incidence, with 30%-40% of patients presenting with stage IV at initial diagnosis, and the 5-year survival rate is below 10% due to limited therapeutic options (2, 3). HAS is a rare and highly aggressive subtype of GC, characterized by a strong tendency to metastasize to the liver and lymph nodes. The 5-year survival rate is approximately 9% (4, 5). Conversion surgery is a therapeutic approach that enables successful tumor resection after aggressive conversion therapy in patients with initially unresectable tumors. In recent years, the combination of chemotherapy and immunotherapy has shown significant efficacy in the treatment of advanced GC, extending OS to 13.1–15.2 months in the general population and to 18.4–19.1 months in patients with a PD-L1 CPS ≥5 (6, 7). Since Yoshida et al. (8) introduced the surgical use of conversion therapy in GC, it has become a key strategy in the management of advanced GC. Accumulating evidence indicates that conversion therapy promotes significant tumor regression, improves the R0 resection rate, and extends OS in patients with advanced GC (9, 10). Nowadays, immuno-chemotherapy has become the first-line treatment option for advanced GC, resulting in a substantially higher probability of tumor regression (6, 11, 12). Shen et al. (13) and Sun et al. (14) each reported a case of HER2-positive advanced HAS successfully converted by chemotherapy. However, follow-up periods were only 2 and 7 months, respectively, and key biomarkers and TRG were not available (NA). Fakhruddin et al. (15) reported a case of HER2-positive advanced HAS achieving 18 months of OS following trastuzumab combined with chemotherapy. Castria et al. (16) described a case of advanced HAS with over 19 months of OS after combined treatment with trastuzumab, ramucirumab, and chemotherapy. Tang et al. (17) reported a case of advanced HAS with a PD-L1 CPS = 20 achieving clinical complete remission after immuno-chemotherapy, with OS exceeding 9 months. Li et al. (18) documented a dMMR advanced HAS patient who achieved pCR with PFS exceeding 6 months after successful conversion therapy with immuno-chemotherapy. Lu et al. (19) reported an advanced HER2-negative, pMMR HAS case with OS exceeding 15 months, although PD-L1 CPS was NA. Although Zhou et al. (20) reported an unresectable cT4aN3aMx HER2-negative, pMMR HAS case with PD-L1 CPS<1 achieving conversion and pCR after three cycles of SOX plus toripalimab conversion therapy, with OS exceeding 12 months, effective treatment options remain elusive for patients with advanced HAS who are HER2-negative, pMMR, programmed cell death protein-1 (PD-1) (note: although we measured PD-1 expression by IHC, it is non-standardized and has not been clinically validated) negative, and PD-L1 CPS<5, and present with both multiple liver and lymph node metastases (**Table 1**).

**Table 1:** Case reports on the conversion therapy and outcomes of all unresectable HAS/AFP-producing gastric cancers from February 2016 to April 2025.

Year	References	Gender	Age (years)	HER2	MMR/MSI	CPS score	Serum AFP	Treatment	Surgical outcomes	RECIST	Follow-up
2016	Shen et al. (13)	Male	70	NA	NA	NA	14,399.9 (ng/mL)	2 cycles of preoperative chemotherapy with CAPEOX; resection of the stomach and external lobe of the left liver; 2 cycles of postoperative chemotherapy with CAPEOX.	R0 resection	NA	PFS >7 months
2016	Sun et al. (14)	Male	66	NA	NA	NA	46.49 (ng/mL)	Preoperative:1cycle with FOLFOX; 4 cycles with DC; 2 cycles with SOX. Radical gastrectomy.	R0 resection	NA	PFS >2 months
2017	Fakhruddin et al. (15)	Female	41	Positive	NA	NA	61,360 (IU/L)	8 cycles with DCX; single agent with trastuzumab for 7 months; 4 cycles with DCX + trastuzumab; 3 cycles FOLFOX with trastuzumab; trastuzumab combined with CPT-11 until patient death.	NA	PD	OS =18 months
2021	Castria et al. (16)	NA	62	Positive	MSS	12	19.568 (ng/mL)	1 cycle with FLOT; 11 cycles with FOLFOX + trastuzumab; single agent with trastuzumab for 2 months; 3 cycles of paclitaxel + lamivudine monoclonal antibody; lamoxirumab maintenance therapy.	NA	PR	OS >19 months
2023	Zhou et al. (20)	Female	48	Negative	pMMR	<1	748.5 (ng/mL)	3 cycles with SOX + toripalimab. Radical gastrectomy.	R0 resection	pCR	PFS >12 months
2024	Bathobakae et al. (45)	Female	49	Negative	NA	NA	1,252 (ng/mL)	EP; death due to intolerance to chemotherapy.	NA	PD	OS = 6 months
2024	Li et al. (18)	Male	64	Negative	dMMR	<1	52,951.56 (ng/mL)	3 cycles with SOX + sintilimab. Total gastrectomy.	R0 resection	pCR	PFS >6 months
2025	Lu et al. (19)	Male	65	Negative	pMMR	NA	4,887.13 (ng/mL)	8 cycles with SOX + tenilizumab; watch-and-wait from December 8, 2023, to July 7, 2024; S-1 + sintilimab for maintenance therapy until disease progression.	NA	MPR	OS >15 months.
2025	Tang et al. (17)	Male	62	Negative	pMMR	20	14,248 (ng/mL)	3 cycles with FP + pembrolizumab; 7 cycles of CAPEOX + pembrolizumab; radiofrequency ablation of the largest intrahepatic lesion + local radiotherapy for hepatogastric lesions + capecitabine and pembrolizumab maintenance.	NA	CR	OS >9 months.

MSI, microsatellite instability; PD, progressive disease; CR, complete response; Search of PubMed/Medline for English language articles using terms (hepatoid adenocarcinomas of stomach) OR (AFP-producing gastric cancer) OR (α-fetoprotein producing gastric cancer) OR (hepatoid esophagogastric adenocarcinoma). Limitation: The current literature on HAS consists predominantly of retrospective case series, with an absence of prospective randomized controlled trials. Inherent to these studies, there was incomplete reporting of HER2/MMR/PD-L1 status, baseline AFP levels, detailed treatment plans, surgical outcomes, pCR status, and follow-up data in a subset of cases.

This study presents a retrospective analysis of a case of HAS with synchronous multiple liver and lymph node metastases. The patient underwent R0 resection following six cycles of conversion therapy, which incorporated immunotherapy in combination with chemotherapy. Postoperative pathological examination confirmed a pCR. It is important to note that this case was marked by a few things: a significantly elevated AFP, HER2-negative, the PD-L1 CPS was 3, PD-1 negative, and pMMR. The conversion regimen proved effective, enabling the patient to achieve R0 resection followed by a pCR. The patient subsequently achieved a PFS exceeding 32 months and an OS of 38 months, indicating a favorable long-term outcome. To our knowledge, this is an exceedingly rare reported case of an advanced HAS patient with HER2-negative, PD-L1 CPS of 3, PD-1-negative, and pMMR status who achieved pCR following conversion therapy with sintilimab combined with Nab-paclitaxel, oxaliplatin, and S-1. Importantly, no recurrence or metastasis has been observed during an OS period exceeding 3 years.

## Case presentation

### Chief complaints

A 51-year-old man was admitted to the hospital with intermittent epigastric pain with melena for 1 month.

### History of present illness

One month prior, the patient experienced acid reflux and belching without an identifiable cause, along with mild epigastric distension, discomfort, and melena. The patient had no other clinical symptoms. Since symptom onset, he had experienced a weight loss of 5 kg.

### History of past illness

The patient has no prior medical history. There was no family history of related conditions or hereditary diseases.

### Physical examination

The patient’s vital signs were stable during the physical examination. No instances of jaundice were observed. No signs of anemia or significant enlargement of superficial lymph nodes were observed. The chest examination yielded no positive findings. The abdomen was flat, with no visible gastrointestinal peristalsis or abnormal contour. The abdomen was soft, with deep epigastric tenderness but no rebound pain. The liver and spleen were not palpably enlarged, although liver percussion tenderness was present. Murphy’s sign and shifting dullness were negative. Bowel sounds were normal, with no abdominal bruits detected. The patient’s BMI was calculated to be 19.13 kg/m², with a body surface area of 1.58 m² and an NRS2002 nutritional score of 3 points. The Eastern Cooperative Oncology Group (ECOG) score was 1.

### Laboratory examinations

**Figure 1:**
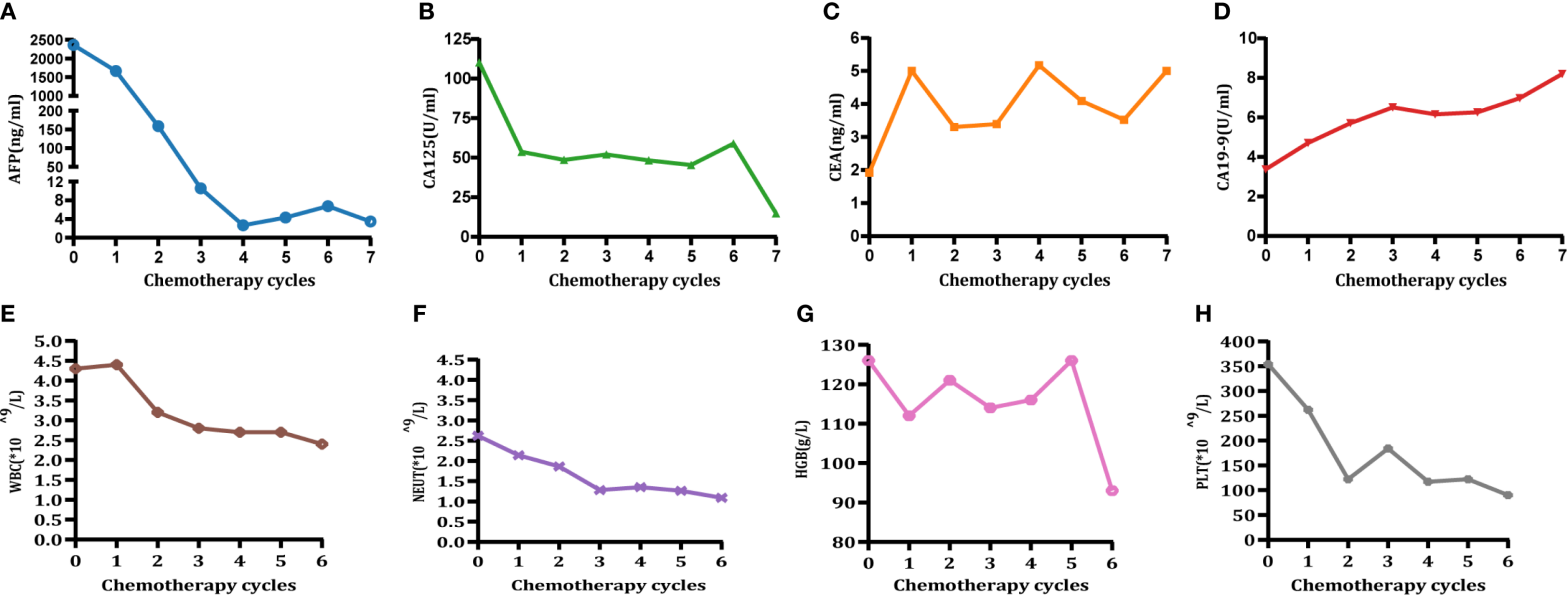
Changes in tumor marker levels **(A-D)** and blood cell count **(E-H)**. Time points: 0 = preoperative level; 1–6 = post-chemotherapy levels for cycles 1 to 6, respectively. **(A)** Changes in AFP (<8.78 ng/mL). **(B)** Changes in CA125 (<35 U/mL). **(C)** Changes in CEA (<5 ng/mL). **(D)** Changes in CA19-9 (<37 U/mL). **(E)** Changes in white blood cell (WBC) count. **(F)** Changes in neutrophil (NEUT) count. **(G)** Changes in hemoglobin (HGB). **(H)** Changes in platelet (PLT) count (CTCAE grading adverse events refer to CTCAE - Version 5.0 (46). Grade 1 hematologic-related AEs occurred after 3-cycle treatment (assessment date: 28 April 2022) and resolved following oral therapy with Leucogen tablets (molecular formula: C14H17O4NS, molecular weight: 295.36) (Leucogen tablets: 20 mg, po, tid). Grade 2 hematological adverse events occurred after 6-cycle treatment (assessment date: 23 July 2022) and resolved following oral therapy with Leucogen tablets.).

Laboratory tests revealed the following values: AFP was 2,359 ng/mL (**Figure 1A**); CA125 was 110.2 U/mL (**Figure 1B**); CEA was 1.92 ng/mL (**Figure 1C**); CA19–9 was 3.71 U/mL (**Figure 1D**); hemoglobin was 126.0 g/L (**Figure 1G**); and a positive fecal occult blood test. All other parameters were within normal limits.

### Gastroscopy and imaging examinations

**Figure 2:**
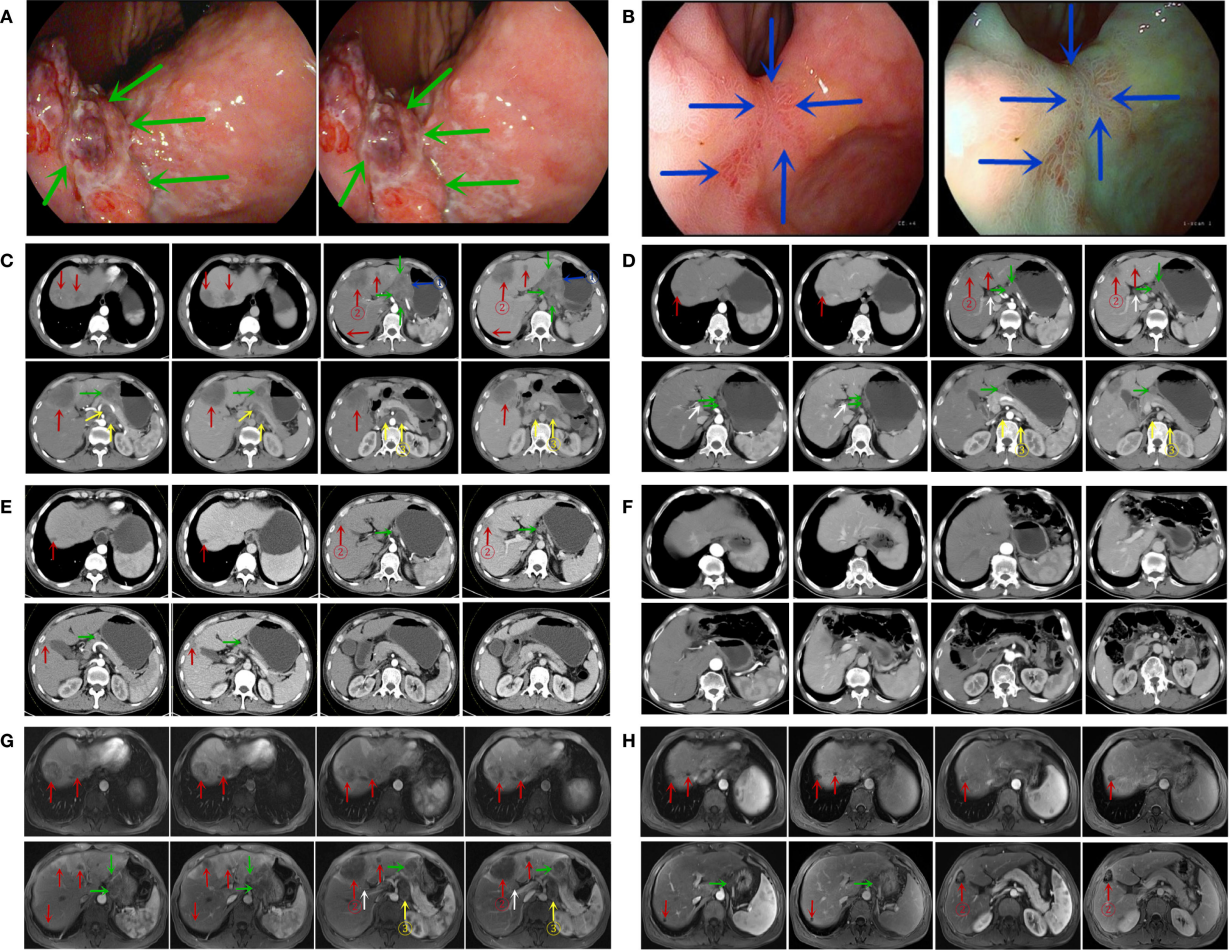
Gastroscopic findings **(A, B)**. **(A)** Gastroscopy at the time of initial diagnosis; **(B)** gastroscopy at the conclusion of six cycles of treatment. (Notes: The green arrow indicates the lesion in the stomach prior to treatment, whereas the blue arrow indicates the lesion in the stomach at the conclusion of the 6-cycle treatment.) CT findings **(C-F)**. **(C)** CT at the initial diagnosis (February 9, 2022); **(D)** CT at the conclusion of three treatment cycles (May 1, 2022); **(E)** CT at the end of six treatment cycles (July 27, 2022); **(F)** CT scan results from the most recent follow-up visit (April 26, 2025). MRI findings **(G, H)**. **(G)** initial MRI at the time of diagnosis (February 9, 2022); **(H)** follow-up MRI after completion of six treatment cycles (July 27, 2022). (The blue arrows indicate the primary gastric lesion, the red arrows indicate liver metastases, the green arrows indicate perigastric lymph nodes, the white arrows indicate hilar lymph nodes, and the yellow arrows indicate retroperitoneal lymph nodes; ① represents the target lesion of the primary gastric lesion; ② represents the target lesion of the liver metastasis; ③ represents the target lesion of the retroperitoneal metastatic lymph node.).

Gastroscopy identified a 2*2.5-cm ulcerative mass at the angular incisure, accompanied by peripheral mucosal erosion and nodular changes. The lesion further extended to involve the lesser curvature (**Figure 2A**, February 8, 2022). Abdominal enhanced CT scan demonstrated local thickening of the anterior gastric wall with ulcer formation, suggestive of ulcerative GC staging T4aN3M1. Multiple liver enhancement lesions were observed indicating metastasis along with enlargement of hepatogastric and retroperitoneal lymph nodes with nodal fusion (**Figure 2C**, February 9, 2022). Abdominal enhanced MRI exhibited multiple abnormal signal shadows in the liver, consistent with metastatic tumors. It also revealed multiple enlarged lymph nodes in both the hepatogastric space and retroperitoneum, indicative of lymph node metastasis (**Figure 2G**, February 9, 2022).

### Final diagnosis

**Figure 3:**
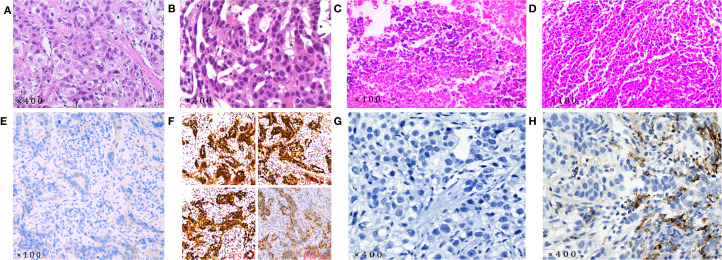
Pathological examination results (all pathological slide examinations were conducted independently, and each slide underwent a secondary review). **(A)** The initial diagnosis revealed gastric adenocarcinoma based on the gastric biopsy results; **(B)** the initial diagnosis was metastatic adenocarcinoma based on the results of the liver FNA biopsy; **(C)** postoperative gastric pathology examination revealed chronic mucosal inflammation; **(D)** no cancer cells were detected in the postoperative liver lesion pathology examination. IHC results. **(E)** HER2-negative; **(F)** pMMR; **(G)** PD-1-negative; **(H)** PD-L1 CPS = 3. (HER-2 antibody: 4B5, with IHC0 and IHC1+ results classified as HER2-negative. The antibodies used for MLH1, MSH2, MSH6, and PMS2 were MX063, MX061, MX056, and MXR019, respectively. Tumors with positive nuclear staining for all four markers are classified as pMMR. PD-1 antibody: MX033. PD-L1 antibody: SP263, with classification based on the CPS: $\text{CPS }=\;\frac{\text{PD }-\text{ L}1\;-\text{ stained tumour cells }+\text{ immune cells}}{\text{total tumour cells}}\;\times \;100$ .).

**Figure 4:**
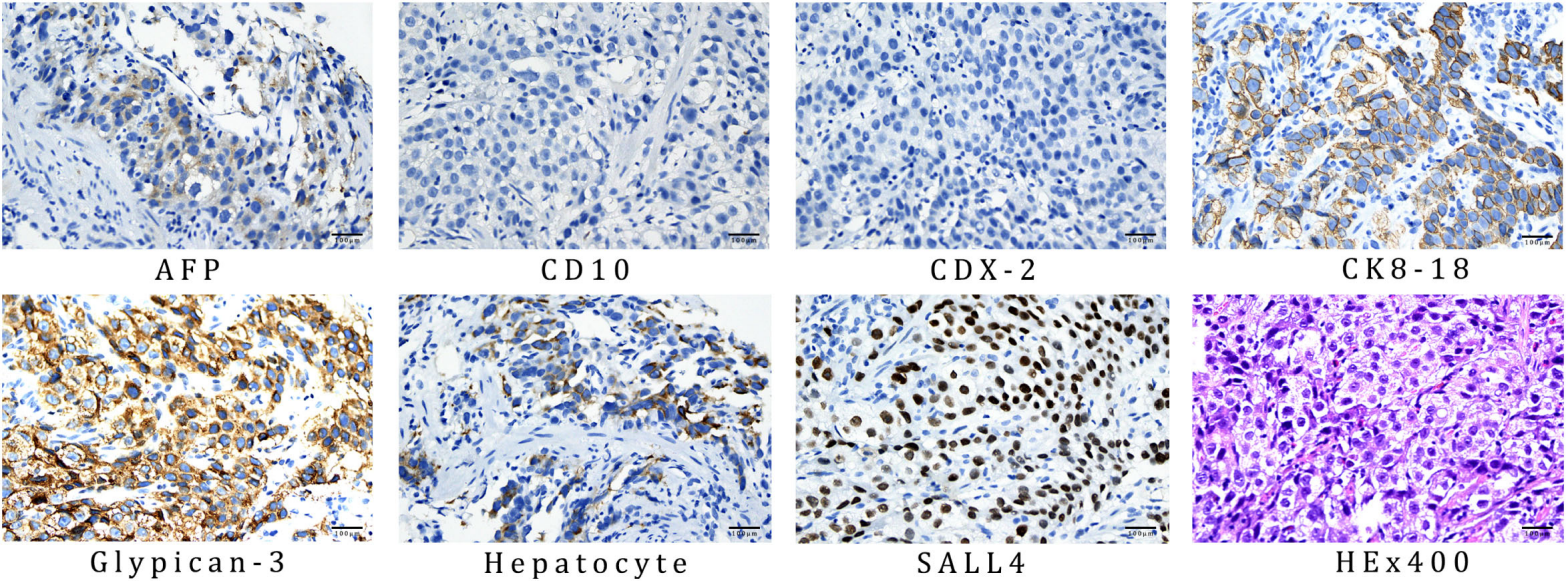
IHC results of gastric biopsy specimens: AFP positive, CD10 negative, CDX-2 negative, CK8–18 positive, glypican-3 positive, hepatocyte positive, and SALL4 positive. The antibodies used for AFP, CD10, CDX-2, CK8-18, glypican-3, hepatocyte, and SALL4 was OTI5D2, MX002, EPR2764Y, 5D3, MAXIM001, OCH1E5, and 6E3, respectively.

The final diagnosis of this patient was HAS combined with liver metastases staged as cT4aN3M1, which corresponds to stage IVB. The endoscopic biopsy confirmed poorly differentiated gastric adenocarcinoma (**Figure 3A**). Further immunohistochemical staining confirmed it to be HAS (**Figure 4**), whereas liver fine-needle aspiration (FNA) biopsy showed hepatic metastases originating from gastric adenocarcinoma (**Figure 3B**). Immunohistochemistry (IHC) revealed HER2-negative (**Figure 3E**), pMMR (**Figure 3F**), PD-1 negative (**Figure 3G**), and PD-L1 CPS = 3 (**Figure 3H**).

### Treatment

The patient received six cycles of conversion therapy involving the administration of sintilimab in combination with Nab-paclitaxel, oxaliplatin, and S-1 (sintilimab: 0.2 g, ivgtt, day 1, q3w; Nab-paclitaxel: 160 mg/m², ivgtt, day 1, q3w; Oxaliplatin: 100 mg/m², ivgtt, day 1, q3w; S-1: 40 mg/m², po, bid, d1–14) (**Table 2**). Throughout the course of treatment, the patient tolerated the chemotherapeutic agents well; Grade 1 hematologic-related AEs occurred after Cycle 3 treatment (assessment date: 28 April 2022) and resolved following oral treatment with Leucogen tablets (molecular formula: C_14_H_17_O_4_NS, molecular weight: 295.36) (Leucogen tablets: 20 mg, po, tid). Grade 2 hematologic-related AEs occurred after Cycle 6 treatment (assessment date: 23 July 2022) and resolved following oral treatment with Leucogen tablets (**Figures 1E-H**), with no non-hematologic AEs. The patient strictly adhered to the treatment protocol and completed each cycle of therapy according to the treatment schedule and oxaliplatin dose adjusted to 150 mg following Grade 1 hematologic-related AEs.

### Outcome and follow-up

After three cycles of conversion treatment, review of enhanced CT (**Figure 2D**, May 1, 2022) compared with pretreatment CT (**Figure 2C**, February 9, 2022), the results showed significant reduction in gastric wall thickening. Liver metastases partially regressed or disappeared, and lymph nodes in the perigastric and retroperitoneal regions showed similar patterns of regression or disappearance. The target lesions tumor regression rates (TRR) for gastric lesions, liver metastases, and retroperitoneal lymph nodes were 42.67%, 79.80%, and 82.53%, respectively (**Table 3**). Serum tumor marker tests suggested decreases in AFP to 10.56 ng/mL (**Figure 1A**), CA125 to 52.0 U/mL (**Figure 1B**), CEA (**Figure 1C**), and CA19-9 (**Figure 1D**) which were stable in the normal range. The disease was evaluated as partial response (PR) [RECIST 1.1 protocol (21)]. However, multidisciplinary team (MDT) discussions concluded that the tumor was still at risk of vascular infiltration and R0 resection was difficult. To maximize the potential for radical surgery and considering the possibility that continued treatment after PR could further reduce tumor burden or achieve pCR, the original conversion therapy regimen was extended for an additional three cycles.

**Table 2:** Timeline of patient care and medication dosing schedule.

Date/medication	Sintilimab (g)	Nab-paclitaxel (mg)	Oxaliplatin (mg)	S-1 (BID) (mg)
1-cycle (2022.02.16)	0.2	250	160	60
2-cycle (2022.03.09)	0.2	250	160	60
3-cycle (2022.04.04)	0.2	250	160	60
4-cycle (2022.05.03)	0.2	250	150	60
5-cycle (2022.05.28)	0.2	250	150	60
6-cycle (2022.06.26)	0.2	250	150	60
2022.08.01	Radical distal gastrectomy + D2 lymph node dissection (No. 1, No. 3, No. 4sb, No. 4d, No. 5, No. 6, No. 7, No. 8a, No. 9, No. 11p, No. 12a) + resection of liver metastases (partial resection of liver segments V, VII, and VIII) + gastroduodenal anastomosis.			
After	Despite the patient’s decision to discontinue further chemotherapy following surgery, follow-up visits were scheduled on a quarterly basis for the initial 2 years, subsequently transitioning to a semestrial frequency. Each subsequent visit incorporated a physical examination, a complete blood count, liver and kidney function tests, and CT scans of the chest, abdomen, and pelvis. During the follow-up period, no CTCAE graded adverse events were documented.			

**Table 3:** TRR and diameter of target lesions following treatment, assessed by RECIST 1.1 (“/” indicates absence of MRI data).

Date/ Examine method/Location/Diameter (cm)	CT (5mm)						MRI (6mm)					
	Gastric lesion: ①		Liver metastasis:②		Retroperitoneal lymph node:③		Gastric lesion: ①		Liver metastasis:②		Retroperitoneal lymph node:③	
	D	d	D	d	D	d	D	d	D	d	D	d
Baseline diameter (Feb 8, 2022)	7.00	2.60	8.00	6.00	4.22	2.55	5.50	2.18	7.20	6.00	4.00	2.00
After 3-cycle (May 1, 2022)	6.00	2.12	4.00	3.81	2.50	1.38	/	/	/	/	/	/
	TRR = 42.67%		TRR = 79.80%		TRR = 82.53%		/		/		/	
After 6-cycle (July 27, 2022)	4.00	1.25	3.00	1.91	< 0.1	< 0.1	3.00	1.00	4.20	2.61	< 0.1	< 0.1
	TRR = 84.49%		TRR = 96.20%		TRR = 100.0%		TRR = 88.43%		TRR = 89.04%		TRR = 100.0%	

$\text{V }=\;\frac{4}{3}\;\pi \;\times \;\frac{\text{D}}{2}\;\times \;{\left(\frac{\text{d}}{2}\right)}^{2}$ (43); $\text{TRR }=\;\frac{{\text{V}}_{0}\;{\text{-V}}_{\text{t}}}{{\text{V}}_{0}}\;\times \;100\%$ (44). V represents tumor volume, D represents the tumor’s maximum diameter, d represents its minimum diameter, V_0_ represents the tumor volume at initial diagnosis, and V_t_ represents the tumor volume at reevaluation following treatment.

Gastroscopy after six cycles of treatment showed an angular incisure ulcer with scarring changes and mucosal congestion (**Figure 2B**, July 26, 2022). Enhanced CT scan (**Figure 2E**, July 26, 2022) showed further shrinkage of gastric lesions. Liver metastases had either partially regressed or disappeared. Similarly, some perigastric lymph nodes had regressed or disappeared, whereas peritoneal lymph nodes were no longer detectable, compared with the previous scan (**Figure 2D**, May 1, 2022). The target lesion TRR for gastric lesions, liver metastases, and retroperitoneal lymph nodes reached 84.49%, 96.20%, and 100%, respectively (**Table 3**). Enhanced MRI scans (**Figure 2H**, July 27, 2022) confirmed that compared with pretreatment (**Figure 2G**, February 9, 2022), the primary gastric tumor had significantly decreased in size. Liver metastases showed partial resolution and partial reduction. Some perigastric lymph nodes decreased in size, whereas others disappeared completely. Retroperitoneal lymph nodes were no longer detectable. The maximum tumor contraction rates were 88.43% for gastric lesions, 89.04% for liver metastases, and 100% for retroperitoneal lymph nodes (**Table 3**). These decreases met the PR criteria of the RECIST 1.1 protocol (21).

Following a consensus by the MDT, the patient underwent a radical distal gastrectomy + D2 lymph node dissection (No. 1, No. 3, No. 4sb, No. 4d, No. 5, No. 6, No. 7, No. 8a, No. 9, No. 11p, No. 12a) + resection of liver metastases (partial resection of liver segments V, VII, and VIII) + gastroduodenal anastomosis on August 1, 2022. Pathology of the postoperative specimen revealed chronic inflammation of the gastric mucosa and reactive hyperplasia of the perigastric lymph nodes (**Figure 3C**). No cancerous cells were detected in the hepatic lesion (**Figure 3D**). The surgical margins of both the stomach and liver were found to be negative. Primary gastric lesions, metastatic liver lesions, and lymph nodes all demonstrated TRG grade 0, thus confirming a pCR (22). After the operation, the patient declined further treatment but continued with regular follow-up. At the 32-month postoperative follow-up, an enhanced CT (**Figure 2F**, April 26, 2025) showed no evidence of tumor recurrence. Serum AFP was 3.49 ng/mL (**Figure 1A**) and CA125 was 14.8 U/mL (**Figure 1B**), and CEA (**Figure 1C**) and CA19-9 (**Figure 1D**) were within the normal range.

### Patient perspective and ethical enrichment

Throughout the treatment, patients and their families were informed about all treatment measures and agreed and signed informed consent forms. The patient had a strong willingness for treatment but was less compliant, especially since no further treatment was administered after surgery. The case report and its accompanying images were published after obtaining written informed consent from the patient.

## Discussion

HAS, a rare GC subtype with histological features mimicking hepatocellular carcinoma (HCC), is the predominant contributor to alpha-fetoprotein-producing GC (AFPGC). Although the incidence of HAS has been reported in the relevant literature to be limited, possibly accounting for only 0.3%-1.0% of all GC (23, 24), with an estimated rate of 0.58-0.83 cases per million people per year (25), it has received increasing attention due to its aggressiveness, especially its susceptibility to hepatic metastasis and poor prognosis (26). Compared with classic GC, HAS had different clinicopathologic features and prognosis than non-HAS. Since the rates of vascular invasion, lymph node metastasis, and liver metastasis were significantly higher in HAS than in non-HAS, the prognosis of HAS was worse than that of non-HAS (P<0.05), and the 5-year survival rate was only 9% (27). A clinical study (28) demonstrated that HAS has unique molecular and diagnostic features, including pMMR, positivity for AFP, salt-like transcription factor 4 (SALL4), overexpression of HER2, PD-L1 CPS, and c-MET, and Epstein–Barr virus-encoded RNA (EBER) negativity. While pMMR and EBER-negative reflect key molecular features of HAS, positivity for AFP and/or positivity for SALL4 are key diagnostic markers for HAS (28). Among these characteristic molecules, patients with HER2-positive or dMMR have a favorable prognosis. AFP-positive GCs show greater aggressiveness and poorer prognosis than AFP-negative ones (29). Moreover, HAS with a high AFP expression (AFP-high HAS) demonstrates significantly worse OS compared with AFP-low HAS (P = 0.046) (30). A survival analysis study showed that preoperative serum AFP levels ≥500 ng/mL were significantly associated with worse OS (p = 0.007) and tended to be associated with worse DFS (p = 0.05) (31). Therefore, an elevated AFP level is an independent prognostic marker for HAS, regardless of liver metastatic status (32). Real-time AFP monitoring holds significant clinical utility, not only in guiding management of advanced GC with elevated AFPGC (33) but also in reinforcing the prognostic relevance of AFP expression across GC subtypes, including HAS (29, 30, 32). Similarly, AFP is an ideal marker for predicting treatment efficacy and determining HAS recurrence. Since AFP expression correlates with tumor cell stemness and immune evasion pathways in HAS, it can be used as a surrogate marker of tumor load and immune response (8, 34). In addition, HAS responds poorly to conventional chemotherapy, leading to a significant shortening of OS and posing a serious threat to patient survival (27). In this case, the patient had a high AFP of 2,359 ng/mL at initial diagnosis and multiple liver metastases and abdominal lymph node metastases, as well as HER2-negative and pMMR, indicating an extremely high degree of malignancy, and the limited effectiveness of conventional treatment to improve the patient’s prognosis. However, the patient exhibited symptoms such as upper abdominal pain and black stools, indicative of a strong desire for treatment and excellent compliance. The patient strictly adhered to the treatment regimen, and his family provided full support and encouragement. AFP rapidly decreased from 2,359 ng/mL to normal levels after four cycles of immuno-chemotherapy and has been maintained within normal levels thereafter. This suggests that the treatment regimen effectively induced tumor regression and reduced tumor viability without evidence of recurrence. These findings are consistent with previous studies, indicating that AFP kinetics correlates with AFPGC and HAS (33, 35).

In recent years, ICIs combined with chemotherapy have shown promise in therapeutic effects in the neoadjuvant treatment of locally advanced CC. This combination can not only improve the R0 resection rate but also increase the pCR rate, thereby improving OS. In the conversion therapy of advanced GC, this strategy improves both the success rate of conversion and the R0 resection and pCR rates, demonstrating a favorable therapeutic effect. A single-arm, phase II clinical study (36) evaluating sintilimab combined with FLOT in patients with HER2-negative, locally advanced adenocarcinoma of the stomach or gastroesophageal junction reported the objective regression rate (ORR) of 84.4% (95% CI, 68.3%-93.1%), the disease control rate (DCR) of 96.9% (95% CI, 84.3%-99.5%), the pCR rate of 17.2% (95% CI, 5.8%-35.8%), the major pathological response (MPR) rate of 55.2%, and the favorable safety profile. A meta-analysis (37) including 21 prospective phase I/II studies with a total of 687 patients reported that immuno-chemotherapy in locally advanced GC resulted in a pCR of 21% (95% CI 0.18-0.24), an MPR rate of 41% (95% CI 0.31-0.52), and an R0 resection rate of 94% (95% CI 0.92-0.96). The incidence of grade 3 or higher AEs was 0.23 (95% CI 0.13-0.38), indicating an overall favorable safety profile (37). Furthermore, Nab-paclitaxel combined with Oxaliplatin and fluorouracil analogs has shown improved therapeutic efficacy and a favorable safety profile in the treatment of GC. FOXAGAST study (38) reported that the combination of Nab-paclitaxel, oxaliplatin, and 5-fluorouracil (FOLFOX) achieved an MPR rate of 38.8% among resectable GC patients, with 16.3% achieving pCR as classified by the Mandard system. The primary toxicities observed were neutropenia, peripheral neuropathy, and nausea; however, most were manageable. These findings suggest that our protocol provided a robust chemotherapeutic foundation for treating this case of HAS. Xia et al. (39) reported a case of advanced GC that received pCR after conversion therapy with Nab-paclitaxel combined with oxaliplatin and an S-1 regimen. Another study, including 147 patients in the final analysis, demonstrated that the DOS (docetaxel, oxaliplatin, and S-1) regimen improved the MPR rate to 25.4% and the R0 resection rate to 78.9% (40).

Shen et al. (13) and Sun et al. (14) each reported one case of advanced HAS that was successfully converted through chemotherapy. However, the follow-up periods were only 2 and 7 months, respectively, and key biomarkers and TRG were NA. A relevant study (28) has shown that HAS patients treated with anti-HER2 therapy or with dMMR have a favorable prognosis. For HER2-positive advanced HAS, trastuzumab combined with chemotherapy may be employed. Fakhruddin et al. (15) documented a case of HAS where the patient achieved an OS of 18 months after treatment with trastuzumab combined with chemotherapy. Castria et al. (16) documented a case of advanced HAS where the patient achieved pCR with successful conversion therapy using trastuzumab, ramucirumab, and chemotherapy, with an OS exceeding 19 months. For HER2-negative advanced HAS, although no ideal targeted therapy exists, immuno-chemotherapy may be considered for dMMR or CPS >5 cases. Tang et al. (17) reported a case of advanced HAS with a CPS of 20 that achieved a clinical complete response and an OS exceeding 9 months following immuno-chemotherapy. Li et al. (18) documented a case of advanced HAS with dMMR that achieved pCR and PFS exceeding 6 months following successful conversion therapy with immuno-chemotherapy. However, there is currently no ideal treatment for HER2-negative, pMMR, CPS<5 cases. Zhou et al. (20) reported a case of advanced HER2-negative, pMMR-type HAS with an undetermined CPS that achieved pCR and PFS exceeding 1 year following successful conversion therapy with immuno-chemotherapy. Lu et al. (19) reported a case of advanced, HER2-negative, pMMR-type HAS with an undetermined CPS that achieved MPR without surgery, with an OS exceeding 15 months. Although an ORR of 58.8% was achieved in a small cohort of AFPGC treated with immuno-chemotherapy (10, 41). However, HAS patients with hepatic metastases face a poor prognosis, with a 5-year survival rate of only 9% (4), with a median OS of only 6–14 months when treated with chemotherapy alone (28, 42). Notably, for patients with advanced HAS with HER-2-negative expression and concurrent multiple liver and lymph node metastases, no effective treatment currently exists. Consequently, these patients face poor survival outcomes, which significantly threaten their prognosis. In our report, the patient declined postoperative treatment but insisted on regular follow-up. The patient, despite being HER2-negative, pMMR, PD-1-negative, and PD-L1 CPS = 3, received immuno-chemotherapeutic conversion therapy. Remarkably, the patient achieved a pCR and survived 32 months postoperatively, with no evidence of recurrence or residual lesions at the last follow-up. In this particular instance, the dosage of Nab-paclitaxel was modified to 160 mg/m², and that of oxaliplatin to 100 mg/m². This regimen was found to maintain therapeutic efficacy while concomitantly reducing the occurrence of dose-dependent AEs associated with the combination therapy. It is worthy to note that the treatment remained effective even subsequent to a reduction in the oxaliplatin dose, on account of Grade 1 hematologic-related AEs. Notwithstanding the presence of pMMR and PD-L1 CPS = 3, sintilimab elicited an immune response, yielding synergistic effects when administered concurrently with chemotherapy. This case suggests that, even in HER2-negative, pMMR, PD-1-negative, and PD-L1 CPS<5 advanced HAS, the combined immuno-chemotherapy offers therapeutic advantages. The following mechanisms may be postulated: firstly, the administration of chemotherapy has been demonstrated to induce immunogenic cell death; secondly, the use of ICIs has been shown to enhance the tumor microenvironment, increase T-cell infiltration, and reverse immune exhaustion by blocking PD-1-PD-L1 binding and its subsequent effects. This results in significant tumor regression and the attainment of substantial therapeutic outcomes.

## Summary

In summary, our patient presented with advanced HAS characterized by extremely high AFP expression, HER2 negativity, PD-1 negativity, pMMR, and PD-L1 CPS of 3, indicating poor response to conventional chemotherapy and a dismal prognosis. However, AFP levels rapidly normalized after six cycles of immuno-chemotherapy conversion therapy. Post-conversion surgical resection confirmed pCR in the primary gastric tumor, hepatic metastases, and lymph nodes, with an OS exceeding 38 months—a finding unprecedented in existing literature. These results suggest that immuno-chemotherapy-based conversion therapy may be an effective strategy for advanced HAS, significantly prolonging survival and potentially achieving curative outcomes.

## Limitations

While this study suggests the potential efficacy of combined immuno-chemotherapy with conversion therapy for advanced HAS, several limitations must be acknowledged. Firstly, as a retrospective case report, it is not possible for it to fully reflect the heterogeneity of the disease. Furthermore, the formulations and dosages of the drugs used cannot yield generalizable conclusions about treatment response patterns. Second, the existing literature on HAS, primarily based on case reports from East Asia (as retrieved from PubMed/Web of Science/Medline for English language articles), may introduce geographical selection bias, making the generalizability of our findings to broader populations uncertain. Furthermore, inherent selection biases and potential confounding factors in treatment selection and disease assessment cannot be ruled out. Therefore, the execution of large-scale prospective cohort studies is imperative to validate our observations, particularly to evaluate the predictive value of biomarkers such as MMR and PD-1/PD-L1. Concurrently, the alternative biomarkers capable of effectively predicting treatment outcomes in cases of pMMR, PD-1-negative, and PD-L1<5 remain to be identified.

## Conclusion

HAS is a highly invasive malignant tumor of the stomach. The dynamic changes in AFP serve as a reliable indicator for detecting HAS, evaluating treatment efficacy, and predicting recurrence. In advanced HER-2-negative, PD-L1 CPS<5, pMMR-type HAS, employing a conversion therapy regimen combining sintilimab with Nab-paclitaxel, oxaliplatin, and S-1 may reduce tumor staging, enhance conversion therapy success rates, and prolong survival.

## Data Availability

The original contributions presented in the study are included in the article/Supplementary Material. Further inquiries can be directed to the corresponding author.
